# Supplementary material for: Identification of photoperiod-induced specific miRNAs in the adrenal glands of Sunite sheep (Ovis aries)
Source: Front Vet Sci. 2022 Jul 22;9:888207. doi: 10.3389/fvets.2022.888207 (PMC9354845; doi:10.3389/fvets.2022.888207)
Supplement: Supplementary file 1 [file Data_Sheet_1.ZIP › Supplementary file/miRDeep2 file.zip!/miRDeep2 file/Novel_Structure/SP42A2/Novel_479.pdf]

Provisional ID	:	NC_019460.2_11636
Score total	:	0.8
Score for star read(s)	:	-1.3
Score for read counts	:	0
Score for mfe	:	0.5
Score for randfold	:	1.6
Score for cons. seed	:	
Total read count	:	25
Mature read count	:	25
Loop read count	:	0
Star read count	:	0

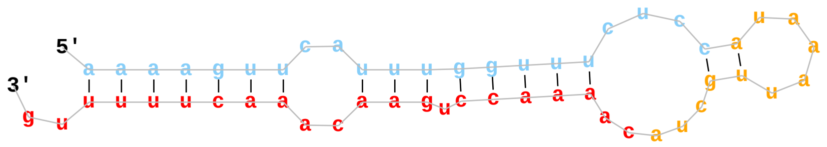

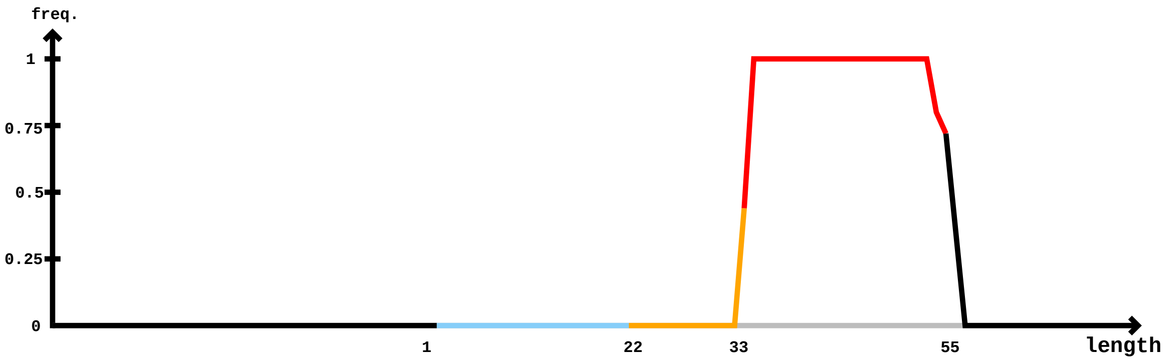

Star Mature

```
5'- agcuggcaugacagcucugcauaauauuggguugaucaaaaaguucauuugguuucuccauaaauugcuacaaaaccugaacaaacuuuuuguccagcucaauacuuggguc -3'    exp
    (((((......)))))..((..(.((((((((((..(((((((((..((((((((...((.....)).....))))).)))..)))))))))..)))))))))).)..)).    reads    mm    sample
    ......................................................................Aaaaaccugaacaaacuuuu.....................    2    1    seq
    ......................................................................Aaaaaccugaacaaacuuuuu....................    1    1    seq
    ......................................................................caaaaccugaacaaacuuuuu....................    1    0    seq
    ......................................................................Aaaaaccugaacaaacuuuuug...................    7    1    seq
    .......................................................................aaaaUcugaacaaacuuuu.....................    2    1    seq
    .......................................................................aaaaccugaacUaacuuuu.....................    1    1    seq
    .......................................................................aaaaccugaacaaacuuuuug...................    4    0    seq
    .......................................................................aaaGccugaacaaacuuuuug...................    1    1    seq
    .......................................................................aaaaccugaaUaaacuuuuug...................    1    1    seq
    .......................................................................aaaaccugaacUaacuuuuug...................    4    1    seq
    .......................................................................aaaaccugaacaaacuuuuugu..................    1    0    seq
```
